# Oral soft tissue disorders are associated with gastroesophageal reflux disease: retrospective study

**DOI:** 10.1186/s12876-017-0650-5

**Published:** 2017-08-07

**Authors:** Masaaki Watanabe, Eiji Nakatani, Hiroo Yoshikawa, Takahiro Kanno, Yoshiki Nariai, Aya Yoshino, Michael Vieth, Yoshikazu Kinoshita, Joji Sekine

**Affiliations:** 1 Department of Oral and Maxillofacial Surgery, Shimane University Faculty of Medicine, 89-1 Enya-cho, Izumo, Shimane 693-8501 Japan; 2 Translational Research Informatics Center, Foundation for Biomedical Research and Innovation, 1-5-4, Minatojima minamimachi, Kobe, Hyogo 650-0047 Japan; 3 Department of Oral and Maxillofacial Surgery, Matsue City Hospital, 32-1 Nosira-cho, Matsue, Shimane 690-8509 Japan; 4 Pathologisches Institut Klinikum Bayreuth GmbH, Preuschwitzer Str. 101, 95445 Bayreuth, Germany; 5 Department of Internal Medicine, Shimane University Faculty of Medicine, 89-1 Enya-cho, Izumo, Shimane 693-8501 Japan

**Keywords:** Dental erosion, Gastroesophageal reflux disease, Gingivitis, Inflammatory oral mucosal regions, Oral soft tissue disorders, Salivary flow volume, Swallowing function

## Abstract

**Background:**

Dental erosion (DE), one of oral hard tissue diseases, is one of the extraoesophageal symptoms defined as the Montreal Definition and Classification of gastroesophageal reflux disease (GERD). However, no study evaluated the relationship between GERD and oral soft tissues. We hypothesized that oral soft tissue disorders (OSTDs) would be related to GERD. The study aimed to investigate the association OSTDs and GERD.

**Methods:**

GERD patients (105 cases), older and younger controls (25 cases each) were retrospectively examined for oral symptoms, salivary flow volume (Saxon test), swallowing function (repetitive saliva swallowing test [RSST]), teeth (decayed, missing, and filled [DMF] indices), and soft tissues (as evaluation of OSTDs, gingivitis; papillary, marginal, and attached [PMA] gingival indexes, simplified oral hygiene indices [OHI-S], and inflammatory oral mucosal regions). Clinical histories, which included body mass index [BMI], the existence of alcohol and tobacco use, and bruxism, were also investigated. A *P* value of <0.05 was defined as statistically significant.

**Results:**

GERD patients, older and younger controls participated and aged 66.4 ± 13.0, 68.3 ± 8.2 and 28.7 ± 2.6 years old, respectively. The most common oral symptom in the GERD patients was oral dryness. Salivary flow volume and swallowing function in the GERD patients were significantly lower than in either of the controls (all *P* < 0.05). Inflammatory oral mucosal regions were found only in the GERD patients. The DMF indices, as a measure of dental caries, in the GERD patients were higher than in the younger controls (*P* < 0.001), but lower than in the older controls (*P* = 0.033). The PMA gingival indexes, as a measurement for gingival inflammation, and OHI-S, as a measure for oral hygiene, in the GERD patients were significantly higher than in either of the controls (all *P* < 0.05). Though no significant differences in BMI, the existence of alcohol and tobacco use were found, bruxism, as an exacerbation factor of periodontal disease, in the GERD patients was significantly more frequent than in either control group (*P* = 0.041).

**Conclusions:**

OSTDs were associated with GERD, which was similar to the association between DE and GERD.

## Background

Salivary flow volume and swallowing function in gastroesophageal reflux disease (GERD) were revealed to be significantly reduced in our preliminary study [1]. Under normal circumstances, saliva and swallowing function have roles in preventing the oral cavity from acid reflux through neutralization and clearing effects [1–3]. According to the Montreal Definition and Classification of GERD, dental erosion (DE) is a major oral symptom caused by acid reflux in patients with GERD [4]. DE is defined as the irreversible loss of dental hard tissue by a chemical process that does not involve bacteria [5, 6]. In our previous study, we found that DE, as an extraoesophageal symptom of GERD, was caused by acid reflux, which resulted in reduced salivary flow volume and swallowing function [1, 4]. Salivary flow and swallowing function may decrease along with oral health condition related to aging [7, 8]. Our preliminary study showed the salivary flow volume and swallowing function in GERD patients were reduced even though considering the effect of aging [1].

The oral cavity consists of teeth, periodontal tissue including gingiva and other oral mucosal regions including the tongue, buccal mucosa, palate, vestibular and oral floor [9–12]. The oral cavity disorders include dental caries, periodontal diseases including gingivitis and periodontitis, and inflammatory oral mucosal regions such as redness, erosion, and ulcer [5, 6, 9–12]. Periodontal disease also as a part results from reduced salivary flow volume [13]. Although we have already revealed the relationships between DE and GERD in our previous preliminary study, complete edentulous patients are included in the GERD patients [1]. Understandably, DE is not in the edentulous GERD patients. We focused on oral soft tissues to detect the edentulous GERD patients from oral findings. Then, oral soft tissue disorders (OSTDs) were defined as gingivitis; one of the periodontal disease, and inflammatory oral mucosal regions. Only a few studies evaluated the relationship between periodontal disease and GERD [14, 15], between oral mucosal inflammation and GERD, separately so far [16, 17]. Furthermore, few studies have evaluated and discussed these co-relationships between OSTDs and GERD, simultaneously. OSTDs are not mentioned as the extraesophageal syndromes of GERD in the Montreal Definition and Classification [4].

We hypothesized that OSTDs would be related to GERD. This retrospective clinical study aimed to evaluate the prevalence of OSTDs in GERD patients in the context of salivary flow volume and swallowing function, to test the above hypothesis. Other general association factors for GERD, including body mass index (BMI), alcohol and tobacco use were also investigated [18–21].

## Methods

### Patient characteristics

The current study was a single-site, cross-sectional retrospective study, and GERD patients and older and younger control subjects were included. GERD outpatients were consecutively selected from the Department of Internal Medicine, Shimane University Hospital between February 2009 and March 2015. A GERD diagnosis was made based in the presence of typical reflux symptoms, such as heartburn and acid regurgitation that occurred more than twice weekly, according to the Montreal definition of GERD [4]. A gastrointestinal fiberscope (GIF) procedure was performed to detect the possible presence of mucosal breaks. GERD patients were divided into two groups, which included a non-erosive reflux disease (NERD) group and a reflux oesophagitis (grades A–D by the Los Angeles classification [22, 23]) group based on the endoscopy findings. As a precondition, our diagnosis of GERD was made in the presence of typical reflux symptoms and finding of GIF. In some cases of GERD, the histopathological examination of the oesophageal mucosa was performed, as necessary. Control groups were outpatients without symptoms or medical histories of gastrointestinal or respiratory system disorders and volunteered in the Department of Oral and Maxillofacial Surgery, Shimane University Hospital, between February 2009 and March 2015. The control group was divided into older and younger subgroups, on the same standard with the preliminary study [1]. The older controls were set as the age-matched with GERD patients. Because aging affects the results of oral examinations, the younger controls were also set [1, 7, 8].

All participants were provided informed consent to participate following approval of the study protocol (No. 398, No. 1082, No. 1217, No. 1750, and No. 1764) by the Ethics Committee of the Shimane University Hospital, Japan.

### Evaluating variables

#### Clinical history

The clinical histories for all participants, which included body mass index (BMI), the existence of alcohol and tobacco use, and bruxism, were retrospectively collected to identify behavioural habits [18–21, 24].

#### Oral cavity complaints in the GERD patients

All participants were interviewed regarding oral cavity complaints before oral examinations regarding oral dryness, acid and bitter taste, glossalgia, halitosis, itching and burning and pharyngeal discomfort.

Four oral examinations, which included salivary flow function, swallowing function, teeth (dental caries), and soft tissues (OSTDs [gingivitis and inflammatory oral mucosal regions]) examinations were performed, as follows;

#### Salivary flow volume

In the Saxon test, the participants were instructed to bite and clamp down on a folded weighed piece of gauze for 2 min. The inserted gauze and a laboratory dish containing the remaining intraoral saliva were then weighed. Salivary flow volume is affected by conditions [25]. The Saxon test was performed twice for each participant, and the average volume of excreted saliva was defined as the salivary flow volume [25]. The saliva was collected under the same standard conditions for each participant.

#### Swallowing function

Swallowing function was evaluated with the repetitive saliva swallowing test (RSST), which assesses the potential to swallow saliva. Swallowing frequency during a 30 s period and time to onset of first swallow were recorded [26]. Swallowing function is also affected by conditions [26]. Therefore, RSST was performed under the same standard conditions for each participant same as salivary flow volume.

### Oral examinations for teeth

#### Dental caries

As examinations for risks of dental caries, the number of remaining teeth, the numbers of decayed (D), missing (M) and filled (treated, F) teeth, the total numbers of DMF (decayed, missing and filled), and DMF indices were used. The DMF index was calculated as follows: total numbers of decayed, treated, and missing teeth/residual teeth × 100 (%) [9].

### Oral examinations for soft tissues

#### Gingivitis

The periodontal disease includes both gingivitis and periodontitis [9–12]. The severity of gingivitis was evaluated to detect GERD from optical findings. As examinations for gingivitis concisely, papillary, marginal, and attached (PMA) gingival indexes for gingival inflammation and simplified oral hygiene indices (OHI-S) scores were used to evaluate oral hygiene. The PMA index scores were defined as the following scores 0: no inflammation, and 1: inflammation of papillary, marginal, and attached gingiva for all teeth [10, 11]. The OHI-S scores included two sub-scores for debris and calculus, each with a possible range of scores from 0 to 3. For OHI-S evaluations, the right upper incisor and left lower incisor and all first molars were evaluated [13, 27, 28].

#### Inflammatory oral mucosal regions

Inflammatory oral mucosal regions, such as the tongue, bilateral buccal mucosa, hard and soft palate, upper and lower vestibular, and oral floor were evaluated [16, 17]. Inflammation severities were classified into “Normal”, “Mild inflammation”, such as redness, and “Severe inflammation” such as erosion or ulcer, from optical findings [16, 17].

#### Relationships between GERD grading and evaluated items in GERD patients

Relationships between GERD grading, NERD and reflux oesophagitis via the Los Angeles classification: (grades A-D), and results of the evaluated variables were compared [23].

#### Comparison between 1) GERD patients and all controls; 2) younger GERD patients and younger controls; 3) older GERD patients and older controls

Finally, parameters regarding the Saxon test, RSST, DMF indices, PMA gingival indexes, and OHI-S between 1) GERD patients and all controls; 2) younger GERD patients and younger controls; 3) older GERD patients and older controls were evaluated and compared. The GERD patients group was also divided into over and under 50 years old conveniently, similar to the older and younger controls.

#### Statistical analyses

A sample size (GERD patients: 105, older controls: 25, and younger controls: 25) was planned and evaluated, which included more patients than our preliminary study (GERD patients: 40, older controls: 15, and younger controls: 15) [1].

Continuous and categorical variables were summarized by mean ± standard deviation (SD) and frequency (and percent), respectively. To compare continuous variables the between two and three groups, Wilcoxon rank-sum test and Kruskal-Wallis test were performed, respectively. Fisher’s exact test was performed for comparison of categorical variables. A *P* value of <0.05 was defined as statistically significant. All statistical analyses were performed using SAS® version 9.3 (Cary, NC, USA) and R version 3.2.2 (R Foundation, Vienna, Austria).

## Results

### Clinical history

GERD patients (105 cases) and older and younger controls (25 cases each) participated. The participants’ information is shown in Table 1. The average for GERD patients, older controls, and younger controls were 66.4, 68.3, and 28.7 years old, respectively. The male proportions of GERD patients, older and younger controls were 54.2%, 48.0%, and 44.0%, respectively. Bruxism in the GERD patients was significantly more frequent than in either control group (*P* = 0.041).

**Table 1:** Participant information

Variable	Category	GERD	Controls	Controls	*P value
		Patients	Older	Younger	
		*n = 105*	*n = 25*	*n = 25*	
Age (years)		66.4 ± 13.0	68.3 ± 8.2	28.7 ± 2.6	NA
Sex	M	57 (54.2%)	12 (48.0%)	11 (44.0%)	0.596
BMI (kg/cm^2^)		22.9 ± 3.6	22.8 ± 4.3	22.1 ± 3.3	0.489
Alcohol use	Yes	29 (27.6%)	6 (24.0%)	3 (12.0%)	0.292
Tobacco use	Yes	19 (18.1%)	3 (12.0%)	3 (12.0%)	0.675
Bruxism	Yes	18 (17.1%)	0 (0.0%)	2 (8.0%)	0.041*

*GERD* Gastroesophageal reflux disease, *NA* Not applied, *M* Male, *BMI* Body mass index

*P values of Kruskal-Wallis test or Fisher’s exact test were determined as significant by *P* < 0.05

### Oral cavity complaints in the GERD patients

The number of GERD patients that had oral cavity complaints was 85 (81.0%). These complaints included oral dryness (59 patients), acid and bitter taste (36 patients), glossalgia (25 patients), halitosis (19 patients), itching and burning (9 patients) and pharyngeal discomfort (1 patient) (multiple answers were allowed). On the other hand, none of the control subjects had any symptoms.

### Salivary flow volume

**Fig. 1:**
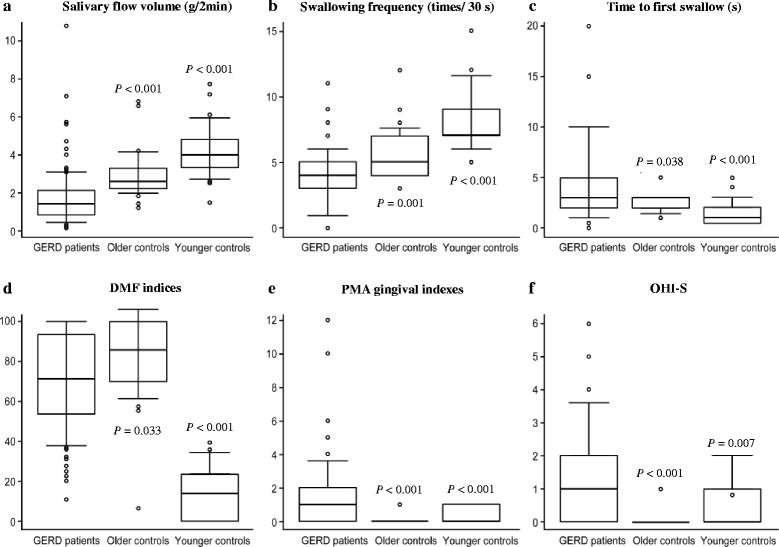
Oral examination comparisons among the GERD patients and the two control groups. **a** Salivary flow volume; Saxon test. The GERD patients had significantly lower levels than older and younger control groups. **b** RSST; Swallowing frequency (times/30 s). The GERD patients had significantly lower levels that the older and younger control groups. **c** RSST; Time to first swallow (s). The GERD patients had significantly longer values than the older and younger control groups. **d** DMF indices. The GERD patients had significantly higher levels than the younger controls, but lower levels than the older controls. **e** PMA gingival indexes. The GERD patients had significantly higher levels than the younger and older control groups. **f** OHI-S. The GERD patients had significantly higher levels than the younger and older control groups. *P values of Wilcoxon rank-sum test were judged as significant by *P* < 0.05

Salivary flow volume, as determined using the Saxon test, in the GERD patients was significantly lower than those in the older (*P* < 0.001) and younger control groups (*P* < 0.001, Table 2 and Fig. 1a).

**Table 2:** Oral examination comparisons between the GERD patient group and the two control groups

Variable	GERD Patients	Older Controls	*P value	Younger Controls	*P value
	*n = 105*	*n = 25*		*n = 25*	
Salivary flow volume; Saxon test (g/2 min.)	1.7 ± 1.5	3.0 ± 1.3	< 0.001*	4.2 ± 1.4	< 0.001*
Swallowing function; RSST					
Swallowing frequency (times/30 s)	4.0 ± 2.1	5.7 ± 2.1	< 0.001*	8.1 ± 2.4	< 0.001*
Time to first swallow (s)	4.9 ± 4.8	2.5 ± 0.9	0.038*	1.6 ± 1.2	< 0.001*
Tooth					
D	1.14 ± 1.75	0.24 ± 0.72	0.003*	0.52 ± 1.26	0.075
M	8.59 ± 8.92	13.84 ± 8.91	0.007*	0	< 0.001*
F	10.2 ± 5.7	8.6 ± 5.8	0.139	3.8 ± 3.4	< 0.001*
DMF	19.9 ± 6.6	22.7 ± 5.8	0.044*	4.3 ± 3.7	< 0.001*
DMF indices (%)	70.2 ± 23.7	80.8 ± 21.1	0.033*	14.9 ± 13.1	< 0.001*
Soft tissues; gingivitis					
PMA gingival indexes	1.58 ± 1.98	0.36 ± 0.49	< 0.001*	0.04 ± 0.20	< 0.001*
OHI-S	1.41 ± 1.45	0.56 ± 0.77	< 0.001*	0.08 ± 0.28	0.007*

*GERD* Gastroesophageal reflux disease, *RSST* Repetitive saliva swallowing test, *D* Decayed, *M* Missing, *F* Filled, *DMF indices* Decayed, missing, and filled indices, *PMA gingival indexes* Papillary, marginal, and attached gingival indexes, *OHI-S* Simplified oral hygiene indices

*P values of Wilcoxon rank-sum test were determined as significant by *P* < 0.05

### Swallowing function

The swallowing frequency in the GERD patients was significantly lower than those in the older (*P* < 0.001) and younger control groups (*P* < 0.001, Table 2 and Fig. 1b). The time to first swallow in the GERD patients was significantly longer than those in the older (*P* = 0.038) and younger control groups (*P* < 0.001, Table 2 and Fig. 1c).

### Oral examinations for teeth

#### Dental caries

The number of remaining teeth ranged from 0 to 32 (mean 20.1) in the GERD patients, from 0 to 32 (mean 14.4) in the older controls, and from 24 to 32 (mean 28.5) in the younger controls. Ten GERD patients were completely edentulous. Additionally, the DMF indices as examinations for risks of dental caries in the GERD patients were significantly higher than in the younger controls (*P* < 0.001), but lower than in the older controls (*P* = 0.033, Table 2 and Fig. 1d).

### Oral soft tissue examinations

#### Gingivitis

The PMA gingival indexes were significantly higher in the GERD patients compared with the younger (*P* < 0.001) and older control groups (*P* < 0.001, Table 2 and Fig. 1e). Additionally, the OHI-S values were also significantly higher in the GERD patients compared with the younger (*P* = 0.007) and older control groups (*P* < 0.001, Table 2 and Fig. 1f).

#### Inflammatory oral mucosal regions

Inflammatory oral mucosal regions were observed in 16 GERD patients (15.2%); however, no regions were observed in either control groups. Inflammation was observed in all mucosal region sites, and the buccal mucosa was the most numerous. Severe inflammations, such as erosion or ulcers, were found in the tongue, bilateral buccal mucosa and soft palate (Table 3). Regarding the oral floor, no inflammation was seen in both GERD and control groups.

**Table 3:** Oral mucosal region inflammation (16 GERD patients)

Oral mucosal region	Total	Mild inflammation	Severe inflammation
Tongue	8	2	6
Right buccal mucosa	4	0	4
Left buccal mucosa	6	2	4
Hard palate	1	1	0
Soft palate	2	1	1
Upper vestibular	1	1	0
Lower vestibular	2	2	0
Oral floor	0	0	0

#### Relationships between GERD grading and the evaluated items

The numbers of NERD and reflux oesophagitis (grade A–D) instances were 62 and 43, respectively. Oral mucosal region inflammation showed a negative correlation; however, there were no relationships between the GERD grading and the other evaluated items (Table 4).

**Table 4:** Associations between GERD grading, which were divided into NERD and reflux oesophagitis (grade A-D) subgroups, and the evaluated items

Variable	Category	GERD *n = 105*			*P value
		Total	NERD *n = 62*	A-D *n = 43*	
Age (years)		66.4 ± 13.0	66.7 ± 10.8	65.9 ± 15.6	NA
Sex	M	57 (54.3%)	38 (61.3%)	19 (44.2%)	0.111
BMI (kg/cm2)		22.9 ± 3.6	22.6 ± 3.4	23.4 ± 3.8	0.294
Saxon test					
Salivary flow volume (g/2 min.)		1.7 ± 1.5	1.5 ± 1.1	1.9 ± 2.0	0.211
RSST					
Swallowing frequency (times/30 s)		4.0 ± 2.1	3.9 ± 2.1	4.2 ± 2.1	0.584
Time to first swallow (s)		4.9 ± 4.8	4.3 ± 3.7	5.7 ± 6.0	0.161
Teeth; DMF indices (%)		70.2 ± 23.7	71.5 ± 22.5	68.5 ± 25.4	0.535
PMA gingival indexes		1.6 ± 2.0	1.5 ± 2.0	1.8 ± 2.0	0.424
OHI-S		1.4 ± 1.5	1.2 ± 1.3	1.7 ± 1.7	0.118
Inflammatory oral mucosal regions	Normal	89 (84.8%)	48 (77.4%)	41 (95.3%)	0.010
	Mild inflammation	6 (5.7%)	4 (6.5%)	2 (4.7%)	
	Severe inflammation	10 (9.5%)	10 (16.1%)	0 (0.0%)	

*GERD* Gastroesophageal reflux disease, *NERD* Non-erosive reflux disease, *M* Male, *NA* Not applied, *BMI* Body mass index, *RSST* Repetitive saliva swallowing test, *DMF* indices: Decayed, missing, and filled indices, *PMA gingival indexes* Papillary, marginal, and attached gingival indexes, *OHI-S* Simplified oral hygiene indices

*P values of Wilcoxon rank-sum test or Fisher’s exact test were determined as significant by *P* < 0.05

#### Comparison between 1) GERD patients and all controls; 2) younger GERD patients and younger controls; 3) older GERD patients and older controls

Regarding the comparison between GERD patients and all controls, both of salivary flow volume and swallowing function in GERD patients were significantly lower than in all controls (*P* < 0.001). DMF indices, PMA gingival indexes and OHI-S in GERD patients were significantly higher than in all controls (*P* < 0.001). Regarding the comparison between younger GERD patients and younger controls, both of salivary flow volume and swallowing function in GERD patients were significantly lower than in all controls (*P* < 0.001). DMF indices in GERD patients were significantly higher than in younger controls (*P* < 0.001). However, no significant differences were seen in PMA gingival indexes and OHI-S. Regarding the comparison between older GERD patients and older controls, salivary flow volume and swallowing frequency were significantly lower than in all controls (*P* < 0.001). Further, no significant difference was seen in time to first swallow and DMF indices. On the other hand, PMA gingival indexes and OHI-S in older GERD patients were significantly higher than in older controls (*P* < 0.001) (Table 5).

**Table 5:** Comparison between 1) GERD patients and all controls; 2) younger GERD patients and younger controls; 3) older GERD patients and older controls

Variable	GERD	All	*P value	Younger GERD	Younger	*P value
	Patients	Controls		Patients	Controls	
	*n = 105*	*n = 50*		*n = 13*	*n = 25*	
Salivary flow volume; Saxon test (g/2 min.)	1.7 ± 1.5	3.6 ± 1.5	< 0.001*	1.8 ± 1.3	4.2 ± 1.4	< 0.001*
Swallowing function; RSST						
Swallowing frequency (times/30 s)	4.0 ± 2.1	6.9 ± 2.5	< 0.001*	4.3 ± 1.3	8.1 ± 2.4	< 0.001*
Time to first swallow (s)	4.9 ± 4.8	2.1 ± 1.1	< 0.001*	4.7 ± 3.4	1.6 ± 1.2	< 0.001*
Teeth; DMF indices (%)	70.2 ± 23.7	47.9 ± 37.6	< 0.001*	50.5 ± 23.9	14.9 ± 13.1	< 0.001*
Soft tissues; gingivitis						
PMA gingival indexes	1.58 ± 1.98	0.2 ± 0.4	< 0.001*	1.20 ± 1.80	0.04 ± 0.20	0.428
OHI-S	1.41 ± 1.45	0.3 ± 0.6	< 0.001*	1.10 ± 1.30	0.08 ± 0.28	0.226
Variable	Older GERD	Older	*P value			
	Patients	Controls				
	*n = 92*					
Salivary flow volume; Saxon test (g/2 min.)	1.7 ± 1.6	3.0 ± 1.3	< 0.001*			
Swallowing function; RSST						
Swallowing frequency (times/30 s)	4.0 ± 2.2	5.7 ± 2.1	< 0.001*			
Time to first swallow (s)	4.9 ± 5.0	2.5 ± 0.9	0.090			
Teeth; DMF indices (%)	73 ± 22.4	80.8 ± 21.1	0.101			
Soft tissues; gingivitis						
PMA gingival indexes	1.6 ± 2.0	0.36 ± 0.49	< 0.001*			
OHI-S	1.5 ± 1.5	0.56 ± 0.77	< 0.001*			

*GERD* Gastroesophageal reflux disease, *RSST* Repetitive saliva swallowing test, *DMF indices* Decayed, missing, and filled indices, *PMA* gingival indexes Papillary, marginal, and attached gingival indexes, *OHI-S* Simplified oral hygiene indices

*P values of Wilcoxon rank-sum test were judged as significant by *P* < 0.05

## Discussion

The medical interview results showed that oral dryness was the most common complaint among the GERD patients (59/105; 56.2%), supporting the findings that GERD patients had a significant risk of suffering from xerostomia [29]. Oral dryness is induced by reduced salivary flow volume [29]. In this study, the salivary flow volume in the GERD patients was significantly reduced, as was observed in our preliminary study [1], which suggested that this was a cause for the oral dryness. All GERD patients included in this study manifested the typical reflux symptoms. Therefore, this result may indicate some correlations between acid reflux as typical GERD symptoms and oral dryness as the atypical symptoms. Swallowing function in the GERD patients was also significantly reduced in this study, as was also observed in our previous study [1].

Dental caries is one of the major oral disorders similar to the periodontal disease [9, 10]. Therefore, the risks of dental caries were also evaluated. As a result, the DMF indices in the GERD patients were significantly higher than in the younger controls, but lower than in the older controls, supporting that idea that dental caries increases with age [9]. Our study revealed no significant relationship between risks of dental caries and GERD.

**Fig. 2:**
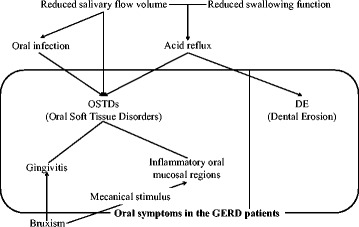
Oral symptoms in the GERD patients. The oral symptoms observed in the GERD patients were DE and OSTDs, which included gingivitis and inflammatory oral mucosal regions. Salivary flow volume and swallowing function were significantly reduced in the GERD patients. OSTDs were induced by damage from gastric acid reflux, similar to DE. The saliva prevents the oral infection by antimicrobial action, cleansing and maintaining mucosal integrity. Therefore, OSTDs were also induced by reduced salivary flow volume. Furthermore, the GERD patients showed a significantly higher frequency of bruxism than the controls; therefore, gingivitis in some GERD patients may be accelerated by bruxism. The bruxism can also be co-cause of inflammatory oral mucosal regions

The PMA gingival indexes levels, as determined by gingival inflammation evaluation items, in the GERD patients were significantly higher than in both control groups. The OHI-S levels, as determined by oral hygiene evaluation items, in the GERD patients were also significantly higher than in both control groups. Saliva protects gingiva by cleaning the tissue and functioning as an antimicrobial agent [14]. Therefore, gingivitis in GERD patients may be induced by reduced salivary flow volume (Fig. 2). Relationships between periodontal disease including gingivitis and GERD have been controversial [14, 15]. The most reasonable explanation for these relationships was reduced salivary flow volume [14].

One of the exacerbation factors of periodontal disease is bruxism, which can increase the rate of progression of pre-existing periodontal disease by mechanically destroying periodontal tissues [30, 31]. Mengatto [32] reported that when a GERD group (*n* = 19) was compared with a non-GERD group (*n* = 26), a statistically higher prevalence of sleep bruxism in the GERD group (14/19; 73.7%) was observed compared with the non-GERD group (6/26; 23.1%, *P* = 0.017). In our present study, the GERD patients (18/105: 17%) showed a significantly higher frequency of bruxism than that of the younger controls (2/25: 8%) and older controls (none). Therefore, gingivitis in some GERD patients may be exacerbated by bruxism (Fig. 2).

It has been reported that sleep bruxism results from acid reflux, occurring via arousal, often together with swallowing [24, 32, 33]. In this study, the bruxism diagnoses were based on participant self-reports, and more detailed objective evaluations, frequency and onset times of the bruxism were then required to demonstrate the concurrence with GERD.

Although the etiology regarding periodontal disease and GERD were not absolutely revealed, the reduced salivary flow volume suggests an association, as it is commonly recognized as one of the exacerbation factors of gingivitis [13, 14]. Furthermore, gingivitis seen in GERD patients can be caused by gastric acid reflux, similar to DE [1] (Fig. 2).

In this study, 10 GERD patients were completely edentulous. In the edentulous patients, we were unable to evaluate the gingiva. However, their oral mucosal regions, except for the gingiva, were evaluated. Inflammatory oral mucosal regions were found only in the GERD patients (16/105; 15.2%). The functions of saliva are antimicrobial action, cleansing and maintaining mucosal integrity [13]. Therefore, inflammatory oral mucosal regions including oral infection, for example, Candida in GERD patients may be caused by reduced salivary flow volume (Fig. 2).

Inflammatory oral mucosal regions in the GERD patients were only reported in the palatal regions, as detected by morphometry [16, 17]. On the other hand, in our present study, inflammation was observed at all sites, including the tongue, buccal mucosa, and palatal regions. Inflammatory oral mucosal regions in the GERD patients may be damaged by gastric acid reflux that spreads into the oral cavity. In particular, inflammation of palatal mucosa can be affected by direct damage from the acid (Fig. 2). Mechanical stimulus by bruxism should be considered. The bruxism is possible to be also co-cause of check-biting [30, 31] (Fig. 2). Especially, the tongue and bilateral buccal mucosa that the most common regions of inflammation were seen in this study, may be affected by the mechanical stimulus, for examples, sharp of teeth edges anatomically. On the other hand, the oral floor may be prevented from the refluxing acid by the tongue and the major salivary glands; sublingal gland, therefore no inflammation was seen [1, 16, 17, 34]. Inflammation was evaluated only optical findings in this study. Therefore it is desirable to be evaluated by scientific methods in the future.

No significant difference was found in any of the evaluated items by GERD grading, which was similar to our preliminary study [1]. Notably, inflammation severity in the oral mucosa and esophagus showed negative correlations. Herbert failed to demonstrate statistical differences between GERD and NERD patients for the prevalence of acidic lesions in the oral cavity and their localization [22]. Though oral and esophagus mucosa is covered by squamous membrane histologically, the degree of keratinization of oral mucosa is completely different from that of esophagus [34]. Especially, the epithelium of the oral mucous membrane is of the stratified squamous variety, which may be keratinized, parakeratinized or nonkeratinized, depending on the location [34–36]. Therefore, the soft tissue damages by acid reflux evaluated in this study, such as redness, erosion and ulcer, may be different between the oral and esophagus region. Other causes of inflammatory oral mucosal regions seen in GERD patients except for the acid reflux, including reduced salivary flow volume and the bruxism may also affect these results.

In this study, the number of cases was enlarged from 40 to 105 in GERD patients, from 15 to 25 in younger and older controls each, in a 5 years sampling interval [1]. Not only the numbers of subjects were enlarged, but also BMI and inflammatory oral mucosal regions were evaluated in addition to our preliminary study [1]. Furthermore, GERD patients were divided into younger and older groups and compared with both controls respectively. According to this result, it could be reconfirmed that salivary flow volume and swallowing function were reduced by not only aging but also GERD. In fact, the older the GERD patients were, the more severe gingivitis might be affected by GERD as a new finding in this study. Therefore, we suggest that OSTDs may be added to DE as the extraesophageal syndromes of GERD.

We recognize some limitations in this study. Firstly, no significant associations in BMI, alcohol and tobacco use were observed. These may be explained by sampling, study design (cross-sectional study), small sample size and less statistical power. Secondly, we did not perform an adjusted analysis with the participant’s background information for comparisons between the GERD patient group and the control groups. Thirdly, we did not consider disease duration and GERD treatment. Thus, in the future, larger prospective studies and detailed analyses are needed to validate our findings. Finally, GERD diagnosis was made on the presence of typical reflux symptoms and GIF without any information regarding pH of saliva. One of the past reports demonstrated significant salivary pH reduction in patients with chronic laryngopharyngitis by GERD before and after treatment [37]. In the future, the correlations with acid exposure, a number of acid reflux events, and potentially proximal extent of reflux should be evaluated objectively.

Our analyses have not addressed the problem for multiple comparisons, and the adjusted a *P*-value was not calculated. However, because the aim of our study is an exploratory study to generate the hypothesis of relationship between GERD and oral hygiene, we believe that we do not need to use the adjusted procedure for multiple comparisons used in confirmatory trials.

## Conclusions

OSTDs were associated with GERD, similar to what was observed for DE.

## Abbreviations

BMI: Body mass index
DE: Dental erosion
DMF indices: Decayed, missing, and filled indices
GERD: Gastroesophageal reflux disease
GIF: Gastrointestinal fiberscope
M: Male
NA: Not applied
NERD: Non-erosive reflux disease
OHI-S: Simplified oral hygiene indices
OSTDs: Oral soft tissue disorders
PMA gingival indexes: Papillary, marginal, and attached gingival indexes
RSST: Repetitive saliva swallowing test
SD: Standard deviation
